# Pictorial Essay: Infants of diabetic mothers

**DOI:** 10.4103/0971-3026.69349

**Published:** 2010-08

**Authors:** Ibrahim A Alorainy, Nauman B Barlas, Amer A Al-Boukai

**Affiliations:** Department of Diagnostic Radiology, King Saud University, College of Medicine and King Khalid University Hospital, Riyadh, Saudi Arabia

**Keywords:** Birth defects, diabetic mothers, maternal diabetes

## Abstract

About 3 to 10% of pregnancies are complicated by glycemic control abnormalities. Maternal diabetes results in significantly greater risk for antenatal, perinatal, and neonatal morbidity and mortality, as well as congenital malformations. The number of diabetic mothers is expected to rise, as more and more of the obese pediatric female population in developed and some developing countries progresses to childbearing age. Radiologists, being part of the teams managing such pregnancies, should be well aware of the findings that may be encountered in infants of diabetic mothers. Timely, accurate, and proper radiological evaluation can reduce morbidity and mortality in these infants. The purpose of this essay is to illustrate the imaging findings in the various pathological conditions involving the major body systems in the offspring of women with diabetes

## Introduction

Improvements in healthcare have resulted in a decline in neonatal problems. The exception is birth defects which have emerged as the most important cause of perinatal loss in diabetic pregnancies.[1] Presently, it is estimated that 3 to 10% of pregnancies are complicated by glycemic control abnormalities and that 80% of these are caused by gestational diabetes.[2] Validated observations suggest significantly greater risk for antenatal, perinatal, and neonatal morbidity and mortality, as well as congenital malformations in infants of diabetic mothers (IDMs).[3,4] A four-fold higher rate of congenital anomalies of brain, heart, kidneys, intestine, and skeleton has been documented in IDMs, suggesting a strong association between congenital anomalies and maternal glycemic control.[4] Current research suggests that the maternal metabolic milieu has a direct influence on the developing embryo during a critical period of organogenesis.[5] Although anomalies in IDMs tend to encompass a spectrum of organ systems rather than result in any specific syndrome, some individual patterns tend to occur more frequently. Thus, major congenital heart disease, musculoskeletal deformities, and central nervous system (CNS) deformities have been the most commonly reported problems[6] [Table 1].

**Table 1:** Congenital anomalies in infants of diabetic mothers

Structural:
Central nervous system
Neural tube defects (meningocele, encephalocele, anencephaly)
Caudal regression syndrome
Holoprosencephaly
Respiratory system
Hyaline membrane disease
Wet lung
Cardiovascular system
Transposition of great vessels
Ventricular and atrial septal defects
Left-sided obstructive lesions (hypoplastic left heart syndrome, aortic stenosis, coarctation)
Genitourinary system
Renal agenesis
Hydronephrosis
Ureteral duplication
Cystic kidneys
Gastrointestinal
Duodenal atresia
Anorectal atresia
Musculoskeletal system
Arthrogryposis
Hypoplastic femur
Functional:
Intraventricular septal hypertrophy
Small left colon syndrome
Others:
Renal vein thrombosis
Adrenal hemorrhage
Macrosomia resulting in difficult vaginal delivery and birth injuries

Gestational diabetes is much more common than nongestational diabetes. The number of diabetic mothers is expected to increase in the future, as more and more of the obese pediatric female population in developed and some developing countries grows to childbearing age. Hence, it is important to address all aspects of this subject. Today, radiologists are part of the teams managing such pregnancies; their role in the detection of congenital anomalies during the antenatal period and in the identification, follow-up, and management of some associated conditions in the perinatal and neonatal period is crucial. It is therefore important for the radiologist to be well aware of the findings that may be encountered in IDMs. Timely, accurate, and proper radiological evaluation can reduce morbidity and mortality in such infants.

The following discussion will address the radiological aspects of abnormalities of the different body systems associated with maternal diabetes.

## CNS and Spine

**Figure 1 (A, B):**
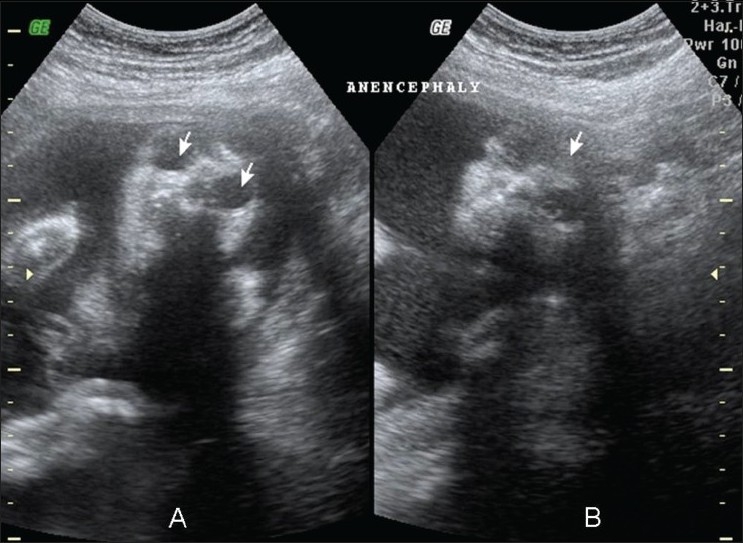
Anencephaly: Prenatal USG done at 18 weeks shows coronal images of the face and orbits with symmetric and complete absence of the cranial vault and brain (arrow in B), above large and prominent orbits (arrows in A)

**Figure 2:**
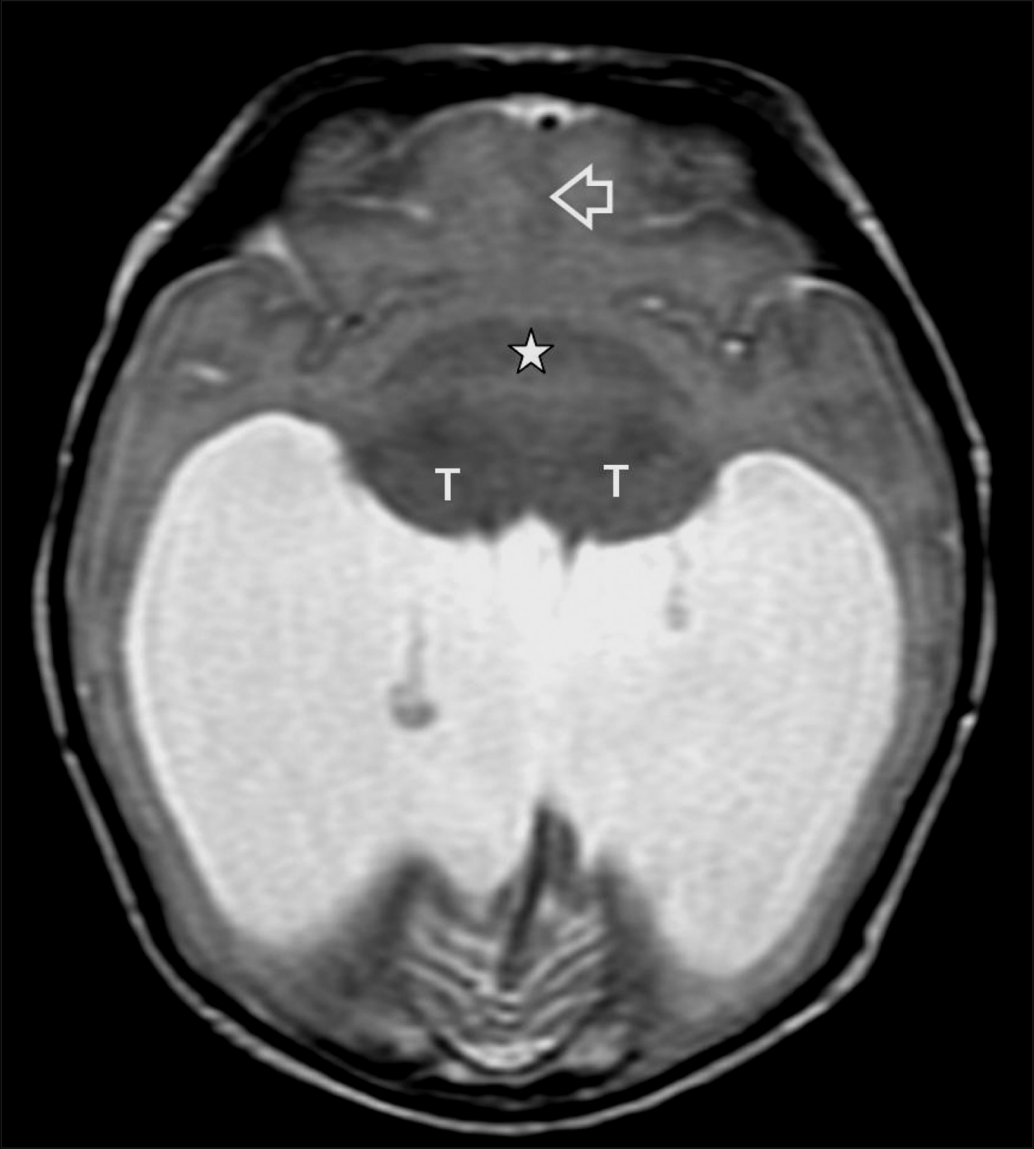
Holoprosencephaly: Axial T2W MRI of the brain at the level of the thalami shows a monoventricle and fusion of the thalami (T), basal ganglia (asterisk), and the frontal lobes, with an absent falx cerebri (open arrow)

**Figure 3 (A,B):**
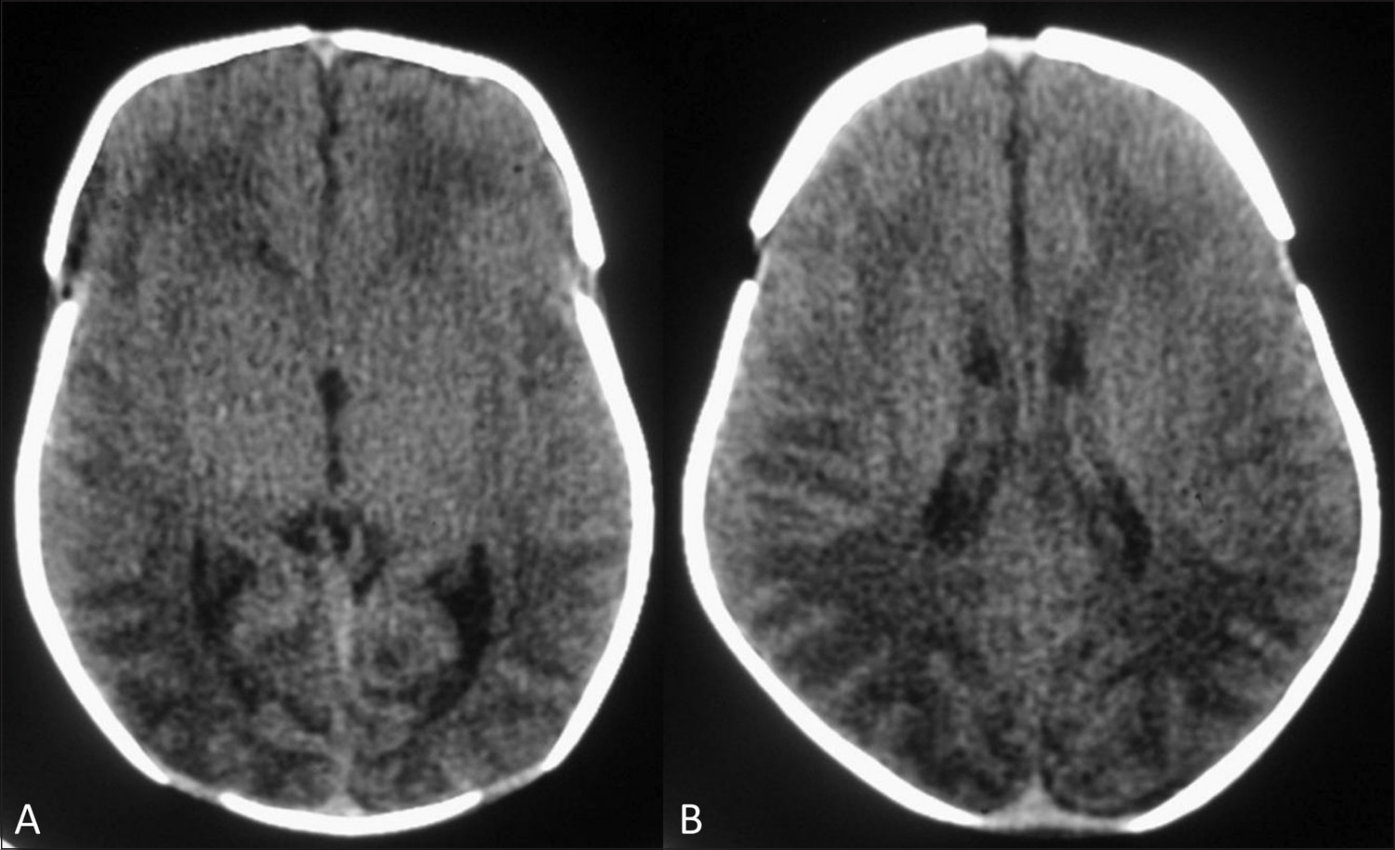
Neonatal hypoglycemia: CT scan of the brain shows typical low attenuation in the white matter of the occipital (A) and parietal (B) lobes on both sides

**Figure 4:**
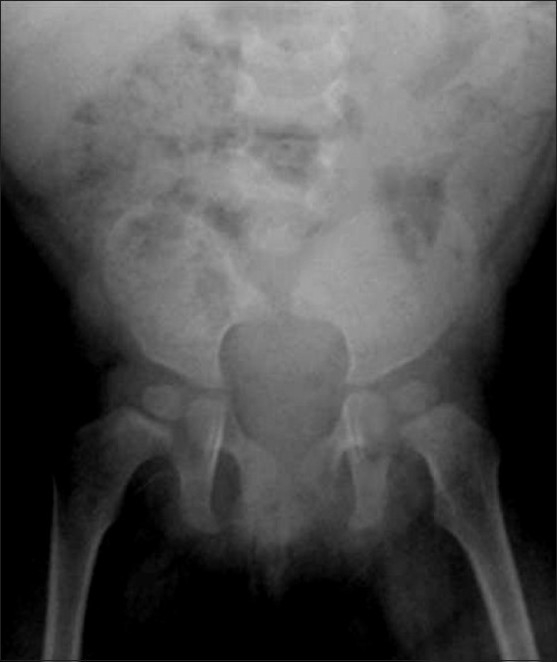
Caudal regression syndrome (complete agenesis of sacrum): Frontal radiograph of the pelvis shows a contracted pelvis due to absence of the sacral segments; the two iliac wings can be seen pseudo-articulating with each other and with the caudal portion of L3 due to the absence of the L4 and L5 vertebrae

The most common structural abnormalities are those related to the failure of neural tube closure and include meningomyelocele, encephalocele, and anencephaly.[4] CNS malformations, particularly anencephaly [Figure 1], open *spina bifida*, and holoprosencephaly [Figure 2] have increased ten-fold in patients with maternal diabetes.[1] Maternal diabetes is one of several conditions that lead to neonatal hypoglycemia [Figure 3]. On the other hand, sacral agenesis or caudal regression syndrome (CRS) [Figure 4], which is the congenital defect thought to be most characteristic of diabetic embryopathy, is seen 200 to 400 times more frequently in IDMs;[7] diabetes must be ruled out in mothers who give birth to infants with sacral agenesis.[8] CRS is also known as caudal dysplasia sequence and is characterized by a series of congenital anomalies[9] [Table 2]. Sirenomelia is considered by some to be an extreme form of the CRS, though the single umbilical, persistent vitelline artery in sirenomelia differentiates the two.[10]

**Figure 5 (A-C):**
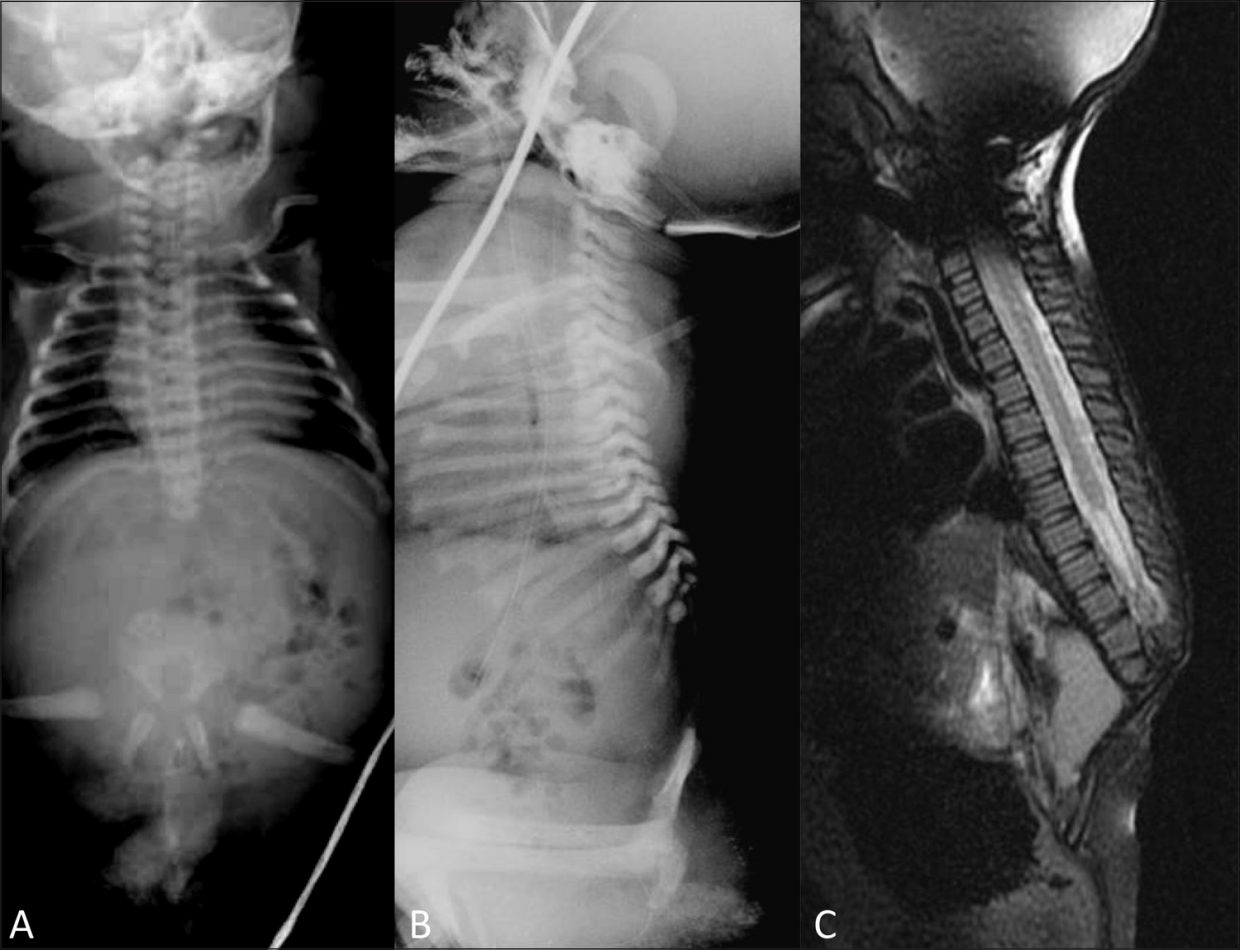
Caudal regression syndrome: Frontal (A) and lateral (B) radiographs of the spine show absence of the sacrum and the entire lumbar spine. The pelvis is very small and the pelvic bones are fused at the midline. Sagittal T2W MRI of the spine (C) shows severe caudal regression with complete absence of the lumbar spine and sacrum. The conus medullaris has a characteristic, abnormal, wedge-shaped (blunted) appearance

Conventional radiographs may demonstrate deformities of the vertebral and pelvic bones as well as of the femur [Figure 4], whereas magnetic resonance imaging (MRI) shows the associated anomalies of the spinal canal, dural sac, and spinal cord [Figure 5].

**Table 2:** Anomalies characterizing caudal regression syndrome

Musculoskeletal anomalies	Complete or partial agenesis of sacrum
	Variable agenesis of lumbar/dorsal spine
	Hypoplastic femur
	Clubbed feet
	Flexion contractures of lower limbs
Spinal cord anomalies	High-positioned wedge-shaped termination of conus
	Tethered cord
	Filar thickening
	Presacral meningocele
	Lipomyelomeningocele
Associated anomalies	Gastrointestinal system
	Anal atresia
	Genitourinary system
	Neurogenic bladder
	Malformed external genitalia
	Renal aplasia

## Respiratory System

**Figure 6 (A,B):**
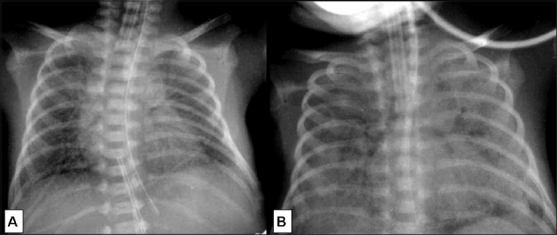
Respiratory distress syndrome (RDS): Portable frontal chest radiograph on day 2 (A) shows reticulogranular opacities throughout both lungs with prominent air bronchograms. The cardiac silhouette is preserved and both lungs are hypoaerated. Frontal radiograph of the chest on day 9 (B) shows more severe changes. There is obliteration of the cardiac and diaphragmatic contours due to marked opacification, giving a complete ‘white out’ appearance

The risk for hyaline membrane disease in IDMs is five- to six-fold greater than in infants of nondiabetic women.[11] Reticulonodular shadowing [Figure 6A] is the hallmark of respiratory distress syndrome (RDS). Hypoaeration and air bronchograms are common. A normal chest radiograph at the age of 6 hours of life virtually rules out RDS.[12] Severe RDS, which may lead to death, is characterized by diffuse alveolar opacities [Figure 6B].

**Figure 7 (A,B):**
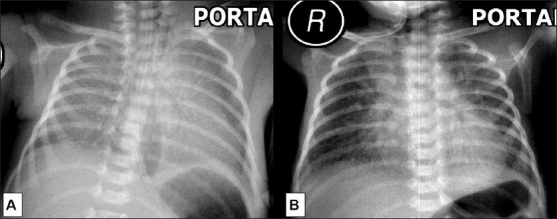
Wet lung (transient tachypnea of the new born): Frontal chest radiograph (A) on day 1 at 6 hours shows a bilateral diffuse ground-glass appearance and fine granularity due to interstitial opacities. Note the left paraspinal lucency which is due to air within the distal esophagus. Frontal chest radiograph (B) of the same patient at the age of 2 days (B) shows that the pulmonary parenchymal opacities have decreased, though perihilar streaky markings are still present

In case of metabolically controlled maternal diabetes, fetal lung maturity is not delayed; however, there is an increased risk of wet lung in the neonatal period.[13] Other conditions that predispose to wet lung are prematurity and precipitous pregnancy. The condition is due to delayed resorption and clearance of fluid from the lung. A chest radiograph obtained within 2 to 6 hours shows mild cardiomegaly, prominent interstitial pattern in the lungs [Figure 7A], and pleural effusion. In contrast to the findings in RDS, the lungs are hyperinflated in wet lung. The condition is self-limiting and clears spontaneously within 2 to 3 days [Figure 7B].

## Cardiovascular System

**Figure 8:**
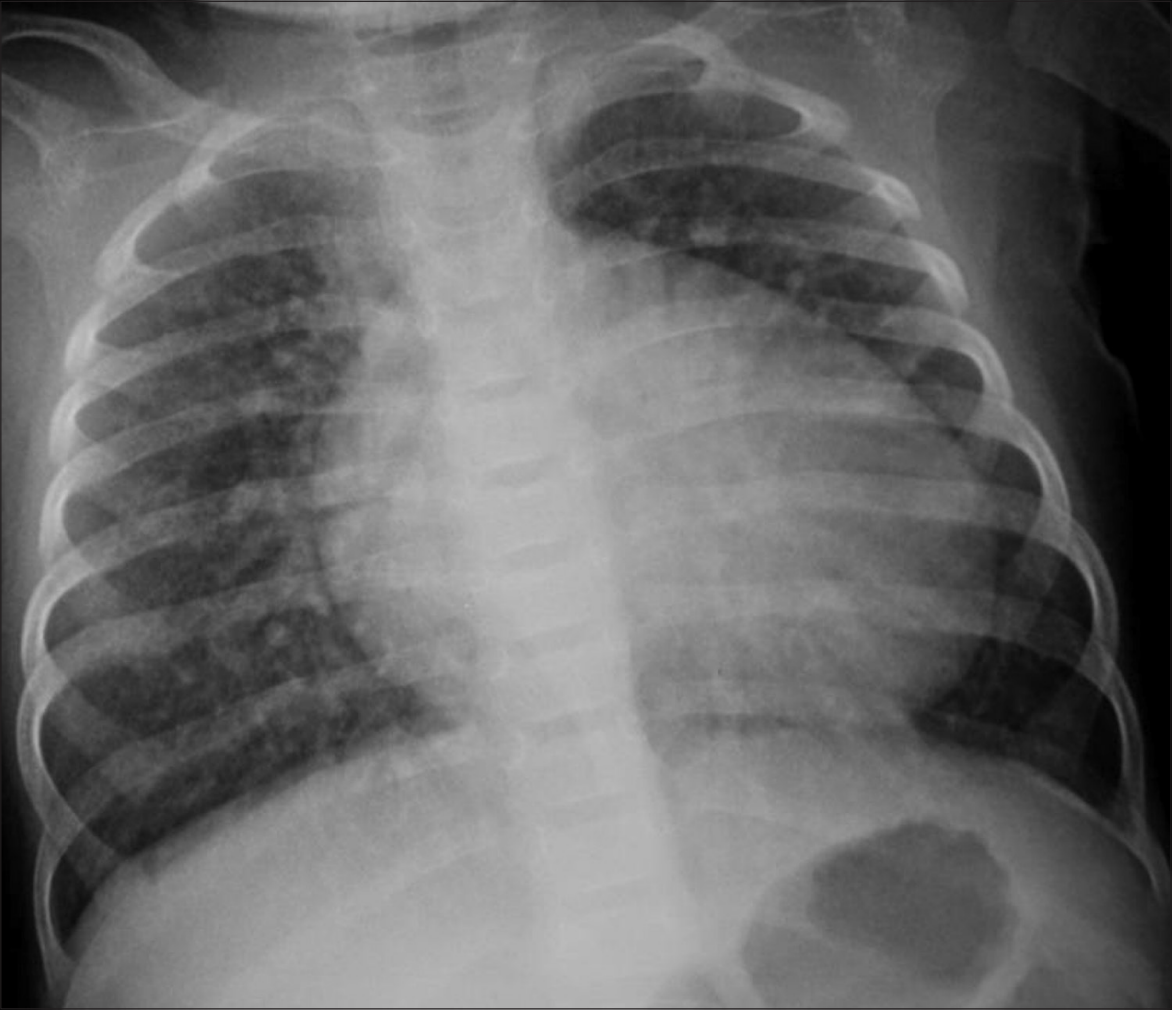
Ventricular septal defect (VSD): Cardiomegaly with plethoric lungs and an inconspicuous aortic knuckle in an infant with ventricular septal defect

Cardiac problems may be structural or functional. Congenital heart disease is about four times more common in IDMs.[8] Structural problems such as transposition of great vessels, truncus arteriosus, and tricuspid atresia are seen three or more times more frequently than expected in IDMs.[14] Wren *et al*.[14] supported the recommendation that all pregnant women with diabetes should be offered a fetal echocardiography, as antenatal diagnosis of such anomalies leads to an improved postnatal outcome. Ventricular septal defects [Figure 8], atrial septal defects, and left-sided obstructive lesions, such as hypoplastic left heart syndrome, aortic stenosis, and coarctation of aorta are other cardiovascular malformations seen in IDMs.[2] Functional abnormalities which are present in up to 30% of IDMs include intraventricular septal hypertrophy and cardiomyopathy; about 10% may have cardiac failure.[15]

## Genitourinary System

**Figure 9:**
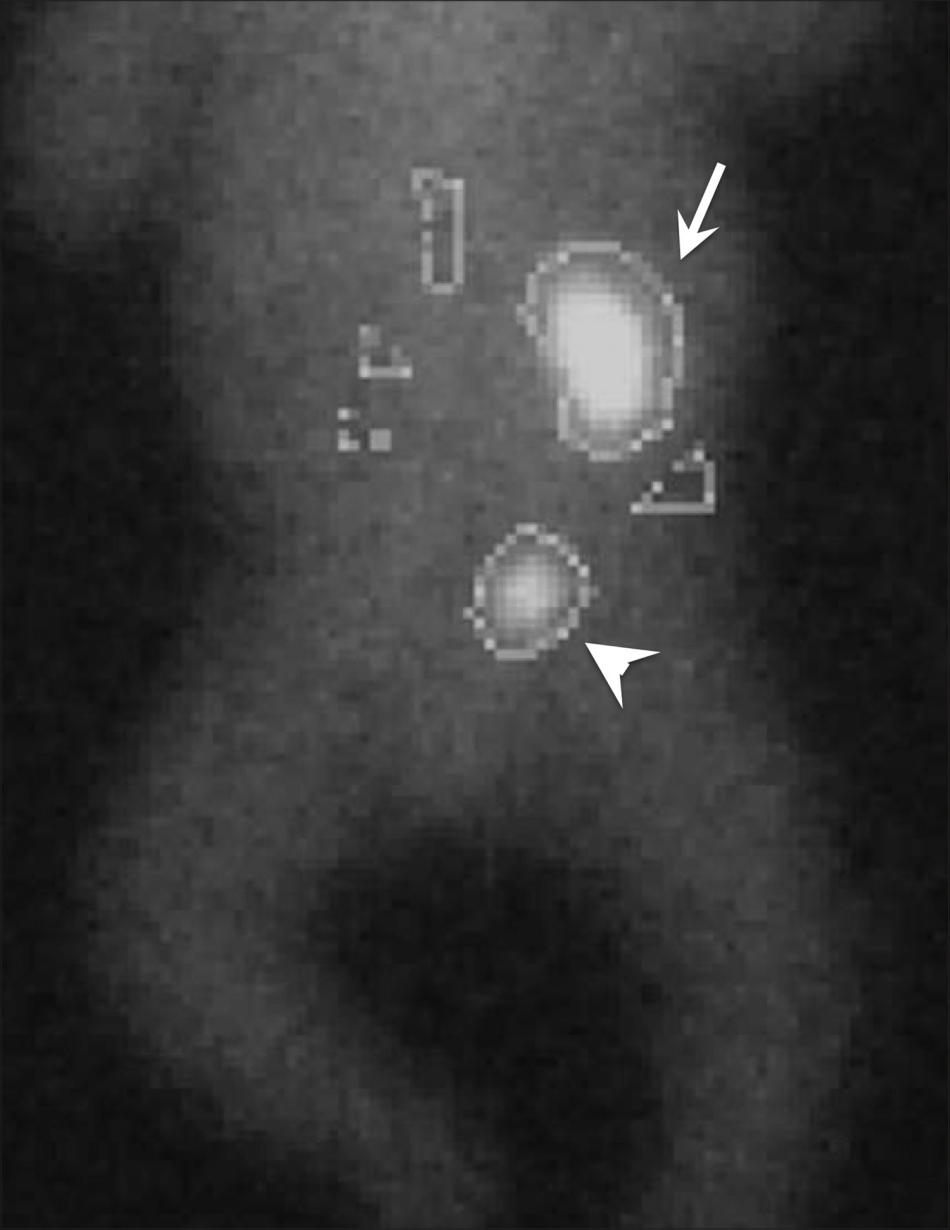
Renal agenesis: DTPA scan shows agenesis of the right kidney. The left kidney and bladder show activity

**Figure 10:**
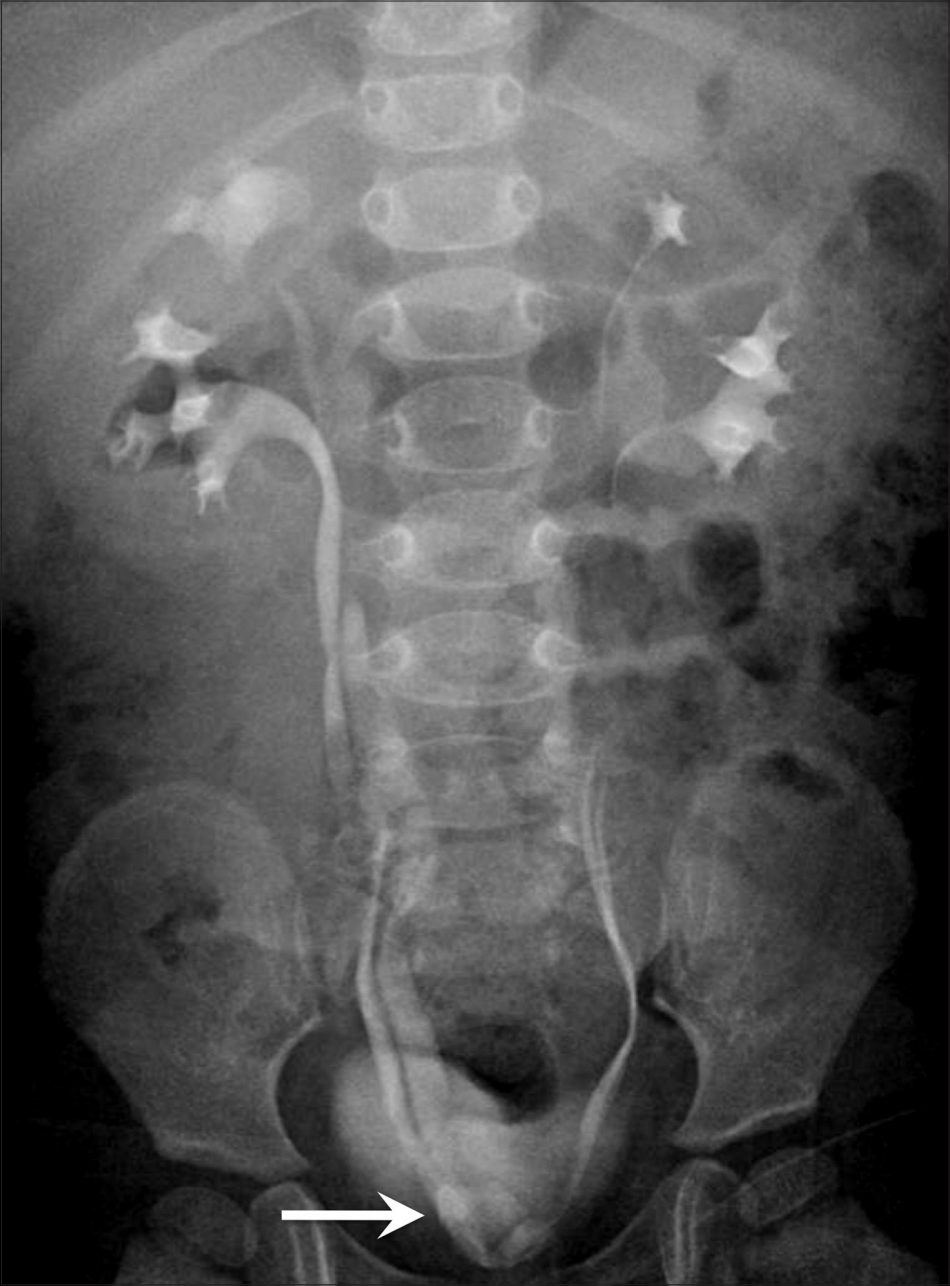
Ureteral duplication: Frontal radiograph from an intravenous urogram study shows bilateral complete ureteral duplication. The opening of the right upper moiety ureter is medial to the lower moiety ureter; there is ureterocele formation

Maternal juvenile diabetes mellitus, nephropathy, hydramnios, and poor metabolic control during a pregnancy complicated by diabetes may result in abnormal development of the fetal kidneys.[16] Several renal anomalies are seen in IDMs, including, renal agenesis [Figure 9], ureteral duplication [Figure 10], hydronephrosis, and cystic kidneys.[15]

A known complication in the genitourinary system in IDMs is renal vein thrombosis which is a severe, life-threatening, but rarely occurring condition.[6] The affected infant presents with a flank mass due to renal enlargement. Color Doppler US may show absence of flow, with loss of pulsation and sometimes a clot in the renal vein.[17]

**Figure 11 (A-C):**
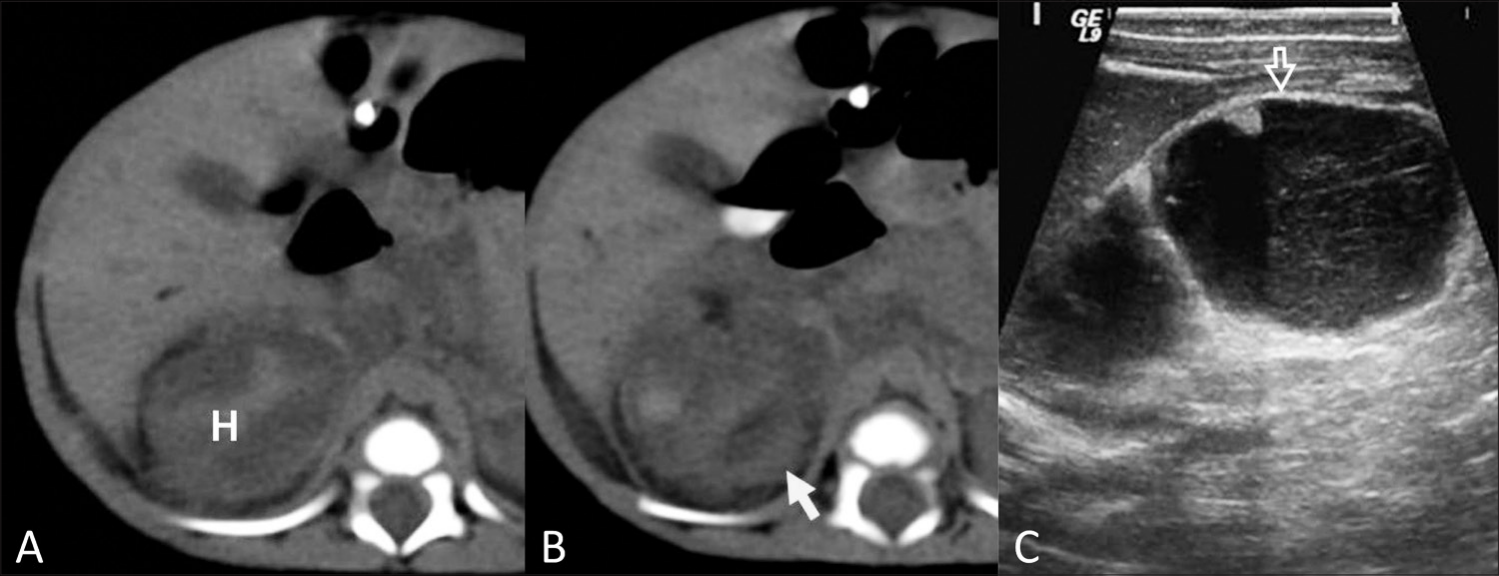
Adrenal hemorrhage: Unenhanced axial CT scans (A,B) at the level of adrenal glands show a heterogeneous mass-like (H) lesion in the right adrenal area with central high density, consistent with blood. The upper pole of the right kidney is seen separately (arrow in B). Follow-up USG for the same patient after 5 weeks shows a complex mass with a mixed, hypoechoic (cystic), and echogenic (solid-like) appearance and peripheral calcific foci (open arrow). The decrease in size suggests resolving hematoma

Adrenal hemorrhage [Figures 11A,B] is also a known entity in IDMs. Large babies such as those seen in diabetic mothers and in the Beckwith-Wiedemann syndrome are predisposed to adrenal hemorrhage. Computed tomography scan and MRI can both diagnose and stage adrenal hemorrhage; however, these modalities usually provide no additional information as compared with ultrasonography (USG) which being free from ionizing radiations can be used in the initial screening and in follow-up.[18]

Early hemorrhage (1 – 2 days), which appears echogenic on ultrasound USG, liquefies shortly thereafter and a cystic or multicystic appearance develops [Figure 11C]. If this does not happen, neuroblastoma should be considered.[19] Calcification [Figure 11C] can also be seen on as sequelae.

### Gastrointestinal system

**Figure 12:**
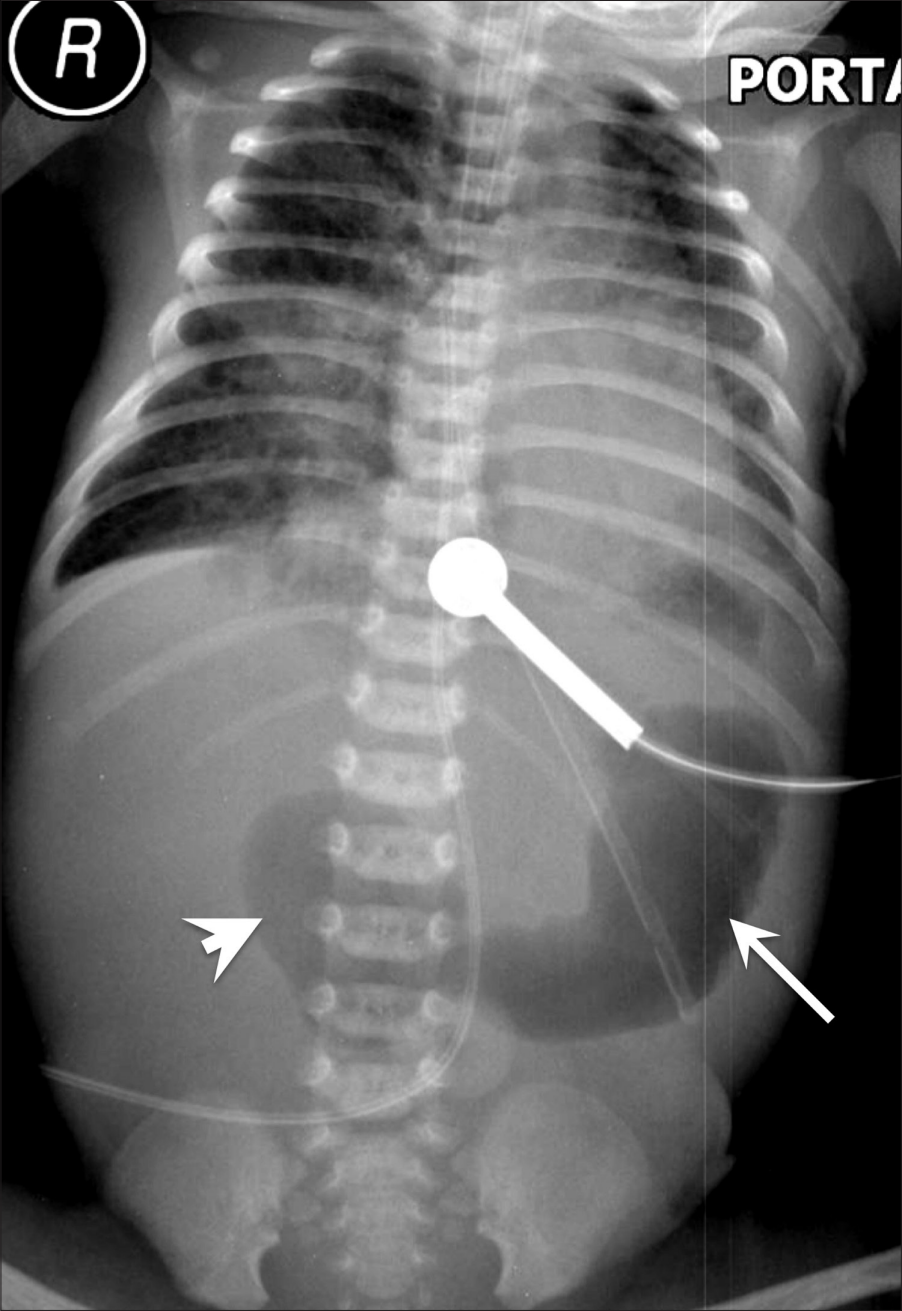
Duodenal atresia: Frontal radiograph of the chest and abdomen on day 2 shows a dilated stomach and proximal duodenum, with the classical ‘double bubble’ sign. Note the absence of gas shadows in the rest of the abdomen and the bilateral patchy basal pneumonia due to aspiration

The most common intestinal anomalies seen in IDMs are atresias of the duodenum and rectum, although atretic segments may be seen at any place along the length of the gastrointestinal (GI) tract. Duodenal atresia is considered to be the most common cause of high GI tract obstruction in IDMs.[20] Abdominal radiograph is usually diagnostic and shows the classic ‘double bubble’ appearance [Figure 12].

**Figure 13:**
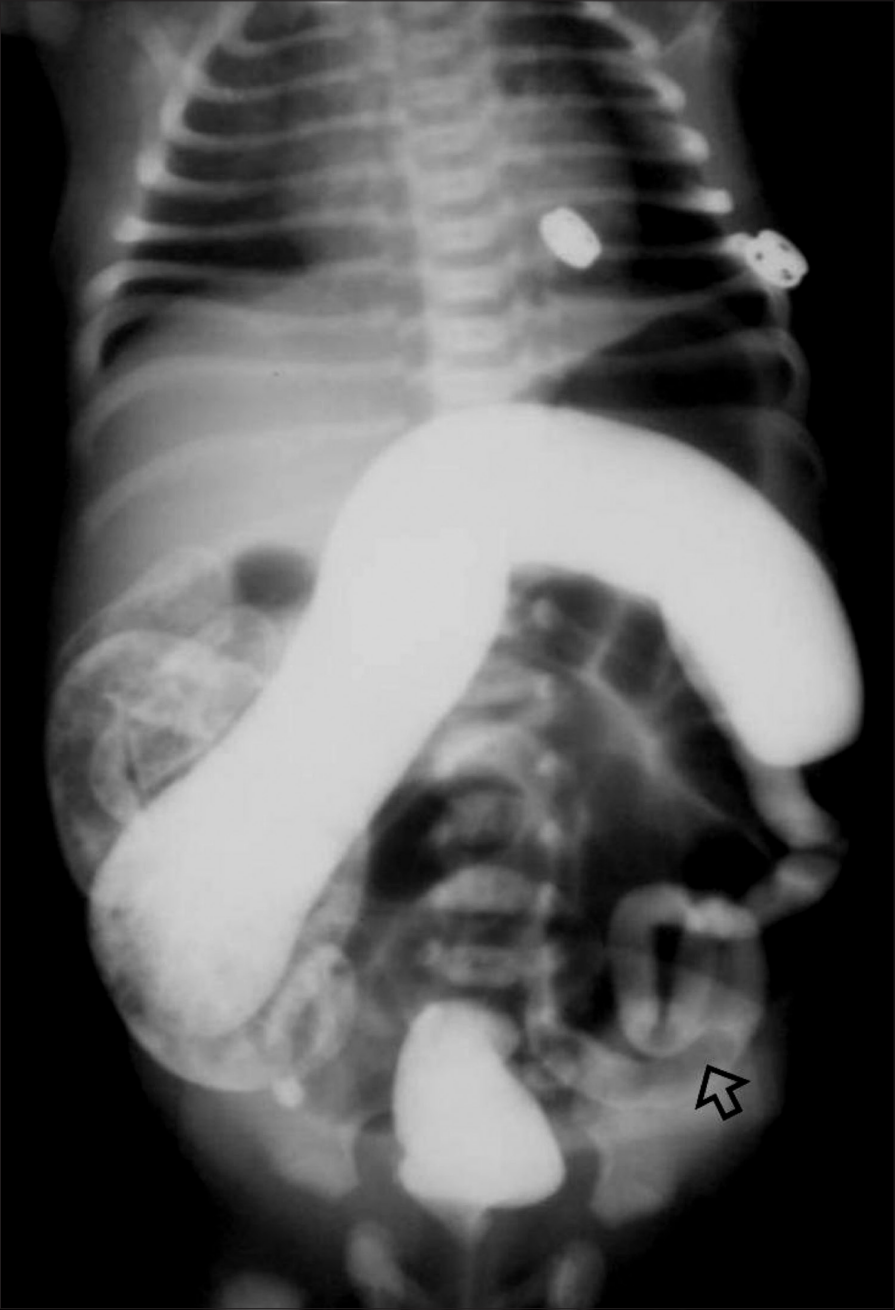
Small left colon syndrome: Frontal radiograph of the abdomen as part of a contrast enema examination using iodinated contrast (gastrografin) shows a narrow caliber of the left colon, with an abrupt transition at the splenic flexure. Multiple filling defects in the left colon are due to meconium plugs (arrow). Also note the rectum is quite distensible

Meconium plug syndrome and small left colon are two overlapping entities in the spectrum of functional neonatal intestinal obstruction.[21] Radiologists use the two term synonymously, or simply the term ‘functional immaturity of colon’ which is a common cause of neonatal distal bowel obstruction in IDMs.[22] In meconium plug syndrome, there is distension of the cecum up to the transverse portion of the colon, with the transition near the splenic flexure; the left-sided colon is narrow and the rectum is quite distensible. Inspissated meconium causes multiple filling defects in the distended portion which may include the distal ileum [Figure 13]. Half of these cases are associated with maternal diabetes. It is important to differentiate meconium plug syndrome from Hirschsprung disease [Table 3].

**Table 3:** Differences between small left colon and Hirschsprung disease

	Small left colon syndrome	Hirschsprung disease
Type of obstruction	Reversible functional	Irreversible functional
Role of contrast enema	Diagnostic and therapeutic	Diagnostic
Rectosigmoid ratio*	More than 1	Less than 1
Appearance of transition zone	Abrupt	Gradual and serrated
Location of transition zone	Splenic flexure	Rectosigmoid junction

* Rectosigmoid ratio = transverse diameter of rectum/transverse diameter of sigmoid colon

**Figure 14 (A,B):**
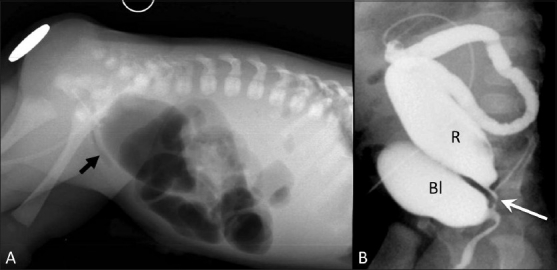
Anorectal malformation: High imperforate anus with recto-uretheral fistula. Cross-table lateral radiograph (A) shows absence of air in the anal area (metallic marker at the anal verge). A lucent curvilinear air shadow is seen anteriorly (arrow) representing air in the urinary bladder as a result of the fistula. Loopogram (B) of the same patient confirms the recto urethral fistula and outlines the rectum and the bladder

Anorectal malformation is another important cause of lower GI tract obstruction in IDMs.[23] The initial abdominal radiograph [Figure 14A] may suggest the level of atresia; however, contrast loopogram [Figure 14B] can confirm not only the level of atresia but also delineate the associated fistula.

### Musculoskeletal system

**Figure 15 (A-C):**
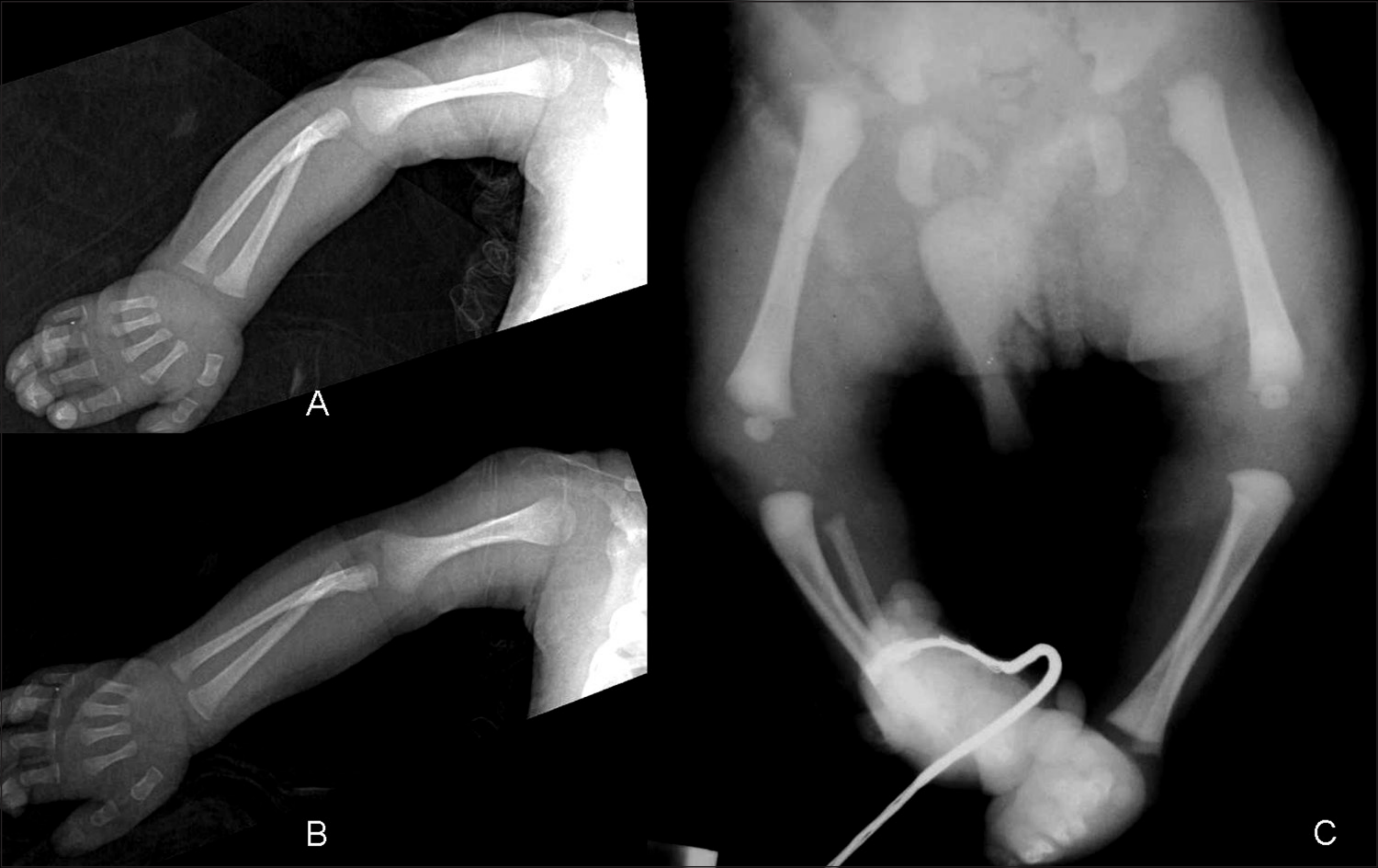
(A–C)Arthrogryposis: Frontal (A) and lateral (B) radiographs of the elbow show extended elbows, pronated forearms, and flexed wrists and fingers in a baby with arthrogryposis. Frontal radiograph of the lower limbs (C) of another baby with arthrogryposis shows bilateral hip dislocations and club feet

**Figure 16:**
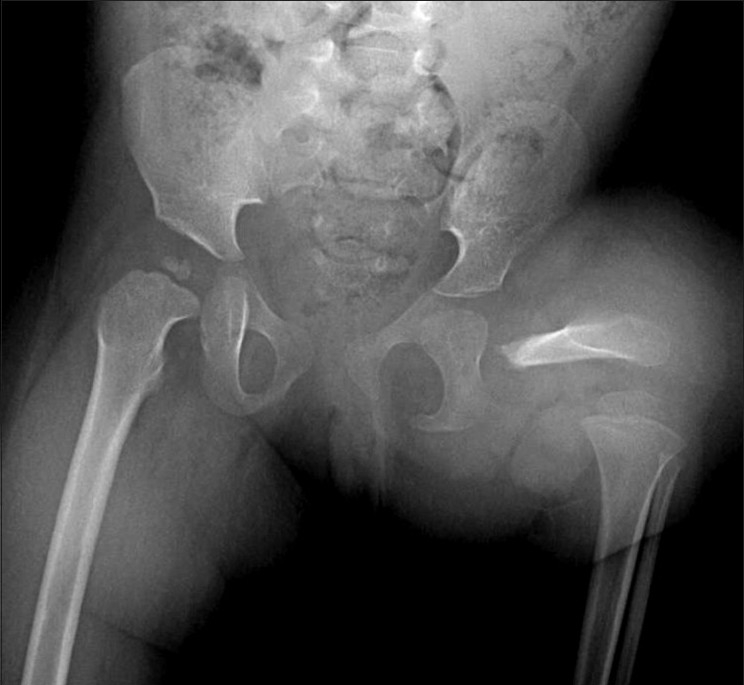
Proximal focal femoral deficiency: Frontal radiograph of the pelvis and both hips shows that the proximal femur is absent and the acetabulum is malformed on the left side. Only a hypoplastic portion of the distal left femur is apparent

Arthrogryposis [Figure 15], hypoplastic femur [Figure 16], and other anomalies of lower limbs are seen more frequently in IDMs.[8] Femoral hypoplasia is a rare anomaly with an estimated incidence of 0.11 to 0.2/10000 live births.[24]

### Fetal growth

Macrosomia is usually defined as a birth weight that exceeds some preselected limit, most frequently 4000 g, or more than 90th percentile for gestational age.[23] Fetal macrosomia is observed in 26% of IDMs and in 8% of infants of nondiabetic women.[23] Fetal macrosomia can cause difficult vaginal delivery due to shoulder dystocia, with resultant birth injuries and asphyxia. These potential birth injuries include cephalhematoma, subdural hematoma, facial palsy, ocular hemorrhage, clavicular fracture, and brachial plexus injuries (Erb palsy). Hepatomegaly, splenomegaly, and cardiomegaly are detectable on plain radiographs.

## Conclusion

There has been significant improvement in the outcome of pregnancies complicated by diabetes mellitus in the last few decades because of the collective efforts of clinicians and researchers. However, there may be a fresh surge of IDMs in the near future as a consequence of the expected increase in the numbers of overweight mothers in the developed as well as some developing countries. Radiologists, being part of the teams managing such pregnancies, should be well aware of the common patterns of anomalies and their appearances on various modalities to play their role in an effective manner.
